# A county-level HIV prevention gap index in the US Deep South using publicly available proxy indicators

**DOI:** 10.3389/fpubh.2026.1793411

**Published:** 2026-04-13

**Authors:** Ruaa Al Juboori, Neva Agarwala, Dylan Barker, Precious Patrick Edet, Brandon Nabors, Alex Vinson, Annina Liebner

**Affiliations:** 1 Department of Public Health, School of Applied Sciences, The University of Mississippi, Oxford, MS, United States; 2 Department of Health Science, South College, Atlanta, GA, United States

**Keywords:** Deep South, Ending the HIV Epidemic (EHE), HIV, HIV testing access, pre-exposure prophylaxis, prevention gap index, spatial epidemiology, viral suppression

## Abstract

**Background:**

The US Deep South bears a disproportionate human immunodeficiency virus (HIV) burden. Mismatch between HIV burden and measurable proxies of biomedical prevention and care performance, pre-exposure prophylaxis (PrEP) utilization, viral suppression (treatment as prevention), and density of listed HIV testing locations may contribute to geographic variation in outcomes, but regional patterns are poorly described.

**Methods:**

We conducted an ecological county-level analysis across the US Deep South states (Alabama, Florida, Georgia, Louisiana, Mississippi, North Carolina, South Carolina, Tennessee, and Texas). Using public datasets, we constructed a Prevention Gap Index (PGI), defined as standardized HIV prevalence minus the mean of three standardized prevention/care proxies aligned with Ending the HIV Epidemic (EHE) domains: pre-exposure prophylaxis (PrEP) (Prevent), viral suppression (Treat), and testing site listing density (Diagnose/access). Indicators were z-standardized, and counties were classified into PGI quartiles to flag potential gaps for planning and resource allocation. Spatial weights used queen contiguity. Multivariable ordinary least squares (OLS) and spatial regression models examined associations between PGI and structural determinants (ethnicity, income, education, and social association measures). Convergent validity was assessed by comparing high PGI counties with EHE Phase I priority counties.

**Results:**

The dataset included 877 counties. Testing site density averaged 7.40 per 100,000 [standard deviation (SD), 8.71]; PGI mean was 0.01 (SD, 1.12). Notably, 65% (13/20) of regional EHE Phase I counties were high PGI (top quartile), and PGI moderately differentiated EHE designation [area under the curve (AUC), 0.761; *p* < 0.001]. Among counties with available new diagnosis rates (*n* = 512), PGI correlated with diagnoses (Spearman coefficient, *ρ* = 0.51; *p* < 0.05) and classified high-diagnosis counties (AUC, 0.819; *p* < 0.001). In bivariate analyses, PGI correlated positively with the percentage of non-Hispanic Black (*r* = 0.362; *p* < 0.001) and negatively with log income (*r* = −0.171; *p* < 0.001), social associations (*r* = −0.095; *p* = 0.005), and some college education (*r* = −0.085; *p* = 0.012). In adjusted OLS models, the percentage of non-Hispanic Black remained positively associated with PGI (*β* = 0.024; 95% confidence interval [CI], 0.020–0.027). Residual clustering supported spatial modeling; spatial lag and error models improved fit, with the spatial error model best capturing remaining dependence.

**Conclusion:**

HIV burden and prevention/care proxies vary spatially across the Deep South. PGI may support geographically targeted planning and monitoring toward national HIV goals and evaluation locally over time, but should be complemented with local program data that capture additional prevention domains.

## Introduction

Four decades into the human immunodeficiency virus (HIV) epidemic, the United States has made important gains in prevention and treatment, yet progress remains uneven across populations and places. Recent surveillance indicates declines in new infections over the past few years. This reflects expanded testing, antiretroviral therapy, and broader prevention options (1–3). However, HIV continues to be concentrated in communities facing persistent structural barriers to prevention, diagnosis, and sustained care (1, 2). At the same time, improved survival has increased the number of people living with HIV. This underscores the importance of long-term engagement in care and viral suppression as both clinical and prevention priorities.

Within this national landscape, the US Deep South remains a major center of HIV burden and ongoing transmission. In this study, we define the US Deep South as Alabama, Florida, Georgia, Louisiana, Mississippi, North Carolina, South Carolina, Tennessee, and Texas (4, 5). This region continues to account for a disproportionate share of diagnoses and prevalent cases (6, 7). Despite major federal initiatives, most notably Ending the HIV Epidemic (EHE), many US Deep South counties continue to lag on key prevention and care indicators, including pre-exposure prophylaxis (PrEP) uptake and viral suppression. Consequently, HIV outcomes remain geographically varied across counties and communities (7, 8).

These regional variations are largely driven by structural and contextual factors than by individual risk alone. Limited insurance coverage, poverty, stigma, and constrained healthcare infrastructure can restrict access to testing, PrEP, and HIV treatment (2, 8). Rurality may shape prevention gaps in complex ways: rural counties often face provider shortages, transportation constraints, and limited local service availability, but the relationship between rurality and prevention mismatch is not necessarily linear because HIV burden and prevention resources are unevenly distributed across the urban–rural continuum (9, 10). Therefore, county-level analysis is needed to identify where the burden outpaces prevention resources.

Spatial epidemiologic research has documented geographic clustering of HIV burden and its co-occurrence with other contextual risks, such as sexually transmitted infection (STI) burden and socioeconomic disadvantage, while prevention resources and uptake may follow different geographic patterns (11–13). Thus far, much of the evidence remains at state or metropolitan scales, which can obscure substantial within-state heterogeneity and limit the identification of specific counties where prevention and care performance proxies are low relative to HIV burden (6, 12).

We situate this work in HIV prevention planning frameworks that emphasize combination prevention and the Ending the HIV EHE pillars, Diagnose, Treat, Prevent, and Respond, with the prevention/care continuum. For county-level planning, three domains are consistently measurable across the US Deep South using publicly available data: (1) diagnosis/service access (availability of HIV testing service locations), (2) biomedical prevention delivery (PrEP utilization), and (3) treatment as prevention performance (viral suppression among people living with diagnosed HIV). Prevention Gap Index (PGI) is not intended to represent a comprehensive latent construct of “prevention capacity”; rather, it summarizes mismatch using a limited set of consistently available county indicators and is intended to flag counties for follow up assessment using local surveillance and program data capturing additional planning domains (e.g., PrEP prescriber capacity and need, testing volume/positivity, linkage/retention, and harm reduction).

County-level incidence/new diagnosis measures would better reflect current transmission intensity, but these are not consistently available across all counties in the region due to suppression and substantial missingness, particularly in lower population counties; therefore, prevalence is used as a service-planning burden metric rather than a proxy for current transmission.

County-level analyses are therefore critical for operational planning because they align with local service catchments, reveal fine grained prevention gaps, and support place-based targeting of prevention and treatment resources. Thus far, most existing studies examine HIV burden or individual prevention indicators in isolation, rather than quantifying where HIV burden is high relative to prevention and care performance proxies across counties. Without an integrated county-level metric, it is difficult to identify “mismatch” counties that may benefit most from targeted prevention expansion.

We introduce PGI as a descriptive screening mismatch metric that summarizes differences between HIV service-planning burden and three consistently available prevention and care performance proxies aligned with EHE domains.

## Methods

### Study design and setting

We conducted an ecological county-level analysis to characterize geographic variation in HIV prevention and care performance proxies across the US Deep South. The US Deep South was defined as Alabama, Florida, Georgia, Louisiana, Mississippi, North Carolina, South Carolina, Tennessee, and Texas (4, 5). The region includes 934 counties/county equivalents. Counties were linked across data sources using Federal Information Processing Standards (FIPS) codes. After linking HIV indicators, testing site data, and county-level covariates, the final analytic sample included 877 counties: Texas had the largest number of counties excluded due to missing HIV burden data (Table 1).

**Table 1:** Pearson correlation analyses of the study variable with PGI, *N* = 877.

Variable	Mean	SD	Correlation	*p*-value
HIV	338.34	269.12	–	–
Viral suppression	67.41	12.06	–	–
Prep	95.91	68.16	–	–
HIV testing service listing density per 100,000	7.40	8.71	–	–
PGI	0.01	1.12		
Health access				
Uninsured	16.054	4.566	0.047	0.1418
Primary care physicians	45.96	27.91	−0.009	0.8005
Socioeconomic factors				
Some colleges	53.243	11.535	−0.085	0.0116
Social associations	9.954	3.676	−0.095	0.0047
Non-Hispanic Black	20.886	18.614	0.362	<0.001
Household income	10.853	0.229	−0.171	<0.001
Broadband access	78.275	8.677	−0.095	<0.01
Rural	0.565	0.294	−0.237	<0.001

### Data sources and measures

County-level HIV indicators were obtained from AIDSVu (Emory University Rollins School of Public Health), which compiles surveillance estimates from the Centers for Disease Control and Prevention (CDC) and state/local health departments (7). We extracted 2023 county measures of: (1) HIV prevalence rate (cases per 100,000 residents; measure of HIV burden), (2) PrEP uses rate, and (3) viral suppression (percentage of persons aged ≥13 years living with diagnosed HIV who were virally suppressed at their most recent viral load test, defined as ≤200 copies/mL). County-level incidence/new diagnosis rates were not used because publicly available county estimates are frequently suppressed or missing across the region, which limits comparability and introduces geographic bias if analyses are restricted to complete case counties.

County-level HIV testing service availability was operationalized as testing service location listing density (listed HIV testing service locations per 100,000 residents) using the HIV.gov services locator API. We queried locations offering HIV testing services, de-duplicated provider records, and spatially joined geocoded locations to county boundaries to aggregate counts by FIPS code. Listing density was calculated as the number of listed testing service locations per county, divided by county population, and scaled to 100,000 residents. To reduce skewness in the data, the measure was log-transformed as ln (1 + sites per 100,000) prior to standardization. This locator-based measure reflects the density of listed service locations (as a proxy for potential geographic availability). It does not capture testing throughput, hours, staffing, service volume, or cross-county travel.

County-level demographic, socioeconomic, and health access indicators were obtained from County Health Rankings and Roadmaps, AIDSVu, and PolicyMap (7, 14, 15). Covariates included uninsured prevalence, primary care physicians per 100,000 population, educational attainment (percentage with some college education), social associations (membership associations per 10,000), median household income, and percentage of non-Hispanic Black. Detailed definitions and source years are provided in Supplementary Table S1.

### Prevention Gap Index (PGI) construction

We constructed a Prevention Gap Index (PGI) as a planning-oriented screening metric that summarizes the mismatch between HIV burden and measurable proxies of county-level biomedical prevention and care system performance. Consistent with EHE planning, we sought county-level measures reflecting actionable domains for geographic prioritization. County measures of prevention capacity (e.g., PrEP prescriber supply, PrEP eligibility/need, testing volume/positivity, linkage/retention, and harm reduction services) are not available consistently across all counties in the nine-state region. We therefore focused on three routinely monitored and publicly available indicators with broad county coverage: PrEP utilization, viral suppression among people living with diagnosed HIV, and the density of locator-based testing service locations. These measures are treated as proxies for prevention and care system performance rather than a measure of capacity; accordingly, PGI is positioned as a screening tool to flag counties for follow-up programmatic review.

PGI is computed from z-scored indicators and is therefore unitless; values represent relative mismatch in standard deviation (SD) units within the analytic sample. We used z-score standardization to place indicators measured on different scales onto a common metric prior to aggregation. The proxy performance composite was calculated as the standardized PrEP utilization, viral suppression, and ln (1 + testing service listing density) indicators. We used equal weights as a transparent default because there is no accepted county-level evidence for assigning relative weights across these domains using cross-sectional public data. Equal weighting should be interpreted as a pragmatic planning assumption for a screening tool, not as evidence that each domain contributes equally to transmission reduction. For a county, *i*, the following is evaluated:

$$\begin{array}{l} \text{Proxy}\;{\text{performance composite}}_{i}\\ =\frac{\begin{array}{l} z({\text{PrEP}}_{i})+z({\text{Viral suppression}}_{i})\\ +z(\ln(1+\text{Sites}\;\mathrm{per}\;100,000_{i}) \end{array}}{3} \end{array}$$

$${\mathrm{PGI}}_{i}=z(\mathrm{HIV}\;{\text{prevalence rate}}_{i})-\text{Proxy}\;{\text{performance composite}}_{i}$$

A 1-unit increase in PGI corresponds to approximately a 1 standard deviation higher burden relative to the proxy performance composite within the nine-state analytic sample. We use prevalence to represent the geographic distribution of people living with diagnosed HIV (service-planning burden); it is not interpreted as a proxy for current transmission intensity. Higher PGI values indicate higher HIV burden relative to prevention and care performance proxies (greater prevention mismatch). Counties were classified as elevated need when PGI was at or above the 75th percentile; elevated need counties were ranked by PGI, with higher ranks indicating greater mismatch. We identified 220 counties in the top quartile of PGI (≥75th percentile cutoff = 0.355). These counties were concentrated in Georgia (*n* = 55) and Texas (*n* = 52), followed by Mississippi (*n* = 29), Louisiana (*n* = 20), South Carolina (*n* = 20), North Carolina (*n* = 18), Alabama (*n* = 11), Florida (*n* = 11), and Tennessee (*n* = 4). Prevention and care performance proxies were computed when at least two of the three prevention indicators were non-missing. Conceptual mapping of PGI components to EHE/continuum domains and key omitted dimensions is provided in Supplementary Table S2.

To avoid relying solely on a single difference score, we also classified counties using a two-axis framework that jointly considers HIV burden and prevention and care performance proxies. Counties were categorized into four quadrants based on high vs. low HIV prevalence (median split) and high vs. low prevention and care proxy performance (median split of the composite prevention capacity score). We emphasize counties in the high-burden/low-capacity quadrant as those most consistent with a “mismatch” interpretation. As z-score difference indices can be sensitive to outliers and distributional assumptions, we evaluated robustness using winsorization and an alternative rank-based scoring approach.

### Sensitivity analyses

We evaluated the robustness of priority county identification to alternative PGI specifications addressing (i) inclusion of viral suppression in the capacity composite and (ii) distributional sensitivity under z-score standardization. Specifically, we recalculated PGI after excluding viral suppression from the prevention capacity score and after winsorizing viral suppression at the 5th–95th percentiles prior to standardization. We also assessed broader outlier handling by winsorizing all component variables [HIV prevalence, PrEP use, viral suppression, and ln (1 + testing service listing density)] at the 5th–95th percentiles prior to standardization. To address conceptual overlap between prevalence burden and viral suppression, we computed an alternative specification (PGI*) using unsuppressed prevalence [prevalence × (1 − viral suppression/100)] as the burden term and excluding viral suppression from the capacity composite. Finally, we examined a rank-based alternative using percentile ranks for all components. We compared overlap in high-need county identification across specifications (top quartile and top decile), summarized in Supplementary Table S3.

Sensitivity analyses indicated that high-need county identification was generally stable across alternative specifications (Supplementary Table S3), including excluding viral suppression from the capacity composite (84.1% overlap in top-decile counties) and winsorization of viral suppression (97.7% overlap in top-decile counties). As longitudinal county-level outcomes suitable for predictive validation (e.g., incidence or subsequent year diagnoses) were not consistently available across the region, we did not conduct predictive validation; however, we conducted convergent validity analyses comparing PGI priority counties with EHE Phase I priority counties. High-need county identification was generally stable across outlier handling and distribution robust alternatives (winsorization and rank-based scoring), which indicates that prioritization was not driven by extreme values or the specific z-score scaling assumptions. Priority county identification was also robust to alternative weighting: top-decile overlap with the equal-weight baseline was 91.9% (PrEP heavy), 90.7% (viral suppression heavy), and 89.5% (testing listing heavy), refer to Supplementary Table S3.

Because modeled incidence is not consistently available and county-level new diagnosis rates are suppressed for many counties, we conducted an exploratory restricted-sample criterion check using AIDSVu 2023 county-level new HIV diagnosis rates where non-suppressed. We assessed Spearman correlation between PGI and PGI* (an alternative PGI specification using unsuppressed prevalence as the burden term and excluding viral suppression from the proxy composite) and diagnosis rates, compared diagnoses distributions across PGI quartiles using Kruskal–Wallis tests, and evaluated discrimination of high-diagnosis counties (top quartile) using logistic regression and AUC. To characterize potential selection effects from suppression, we compared county population size between counties with suppressed and non-suppressed diagnosis rates. We also evaluated the sensitivity of PGI to the equal-weight assumption by recalculating the proxy performance composite under three alternative weighting scenarios in which one proxy received 50% weight, and the remaining two proxies received 25% each (PrEP-heavy, viral suppression-heavy, and testing-listing-heavy). We compared top-decile priority county membership under each scenario to the equal-weight baseline using the percentage of overlap (baseline capture).

### Statistical analysis

We summarized study variables using means and standard deviations. Pearson correlation coefficients were computed to describe bivariate associations between PGI and candidate determinants. Multivariable ordinary least squares (OLS) regression models were used to evaluate associations between PGI and structural determinants. Statistical significance was assessed using two-sided tests with *p* < 0.05. OLS coefficients are reported with 95% confidence intervals (CIs), and *p*-values.

### Spatial analysis

We mapped county-level distributions of HIV prevalence, prevention indicators, PGI, and key determinants across the US Deep South. For multivariable modeling, we tested for residual spatial autocorrelation in the OLS model using Moran’s I on the OLS residuals, using the same spatial weights as the spatial models. As residuals exhibited spatial dependence, we estimated spatial regression models, spatial lag (SAR) and spatial error (SEM), using queen contiguity spatial weights that were row standardized. Model fit was compared using information criteria and residual spatial diagnostics. For SAR, we report the spatial autoregressive parameter (*ρ*); for SEM, we report the spatial error parameter (*λ*). SAR/SEM coefficients are reported with 95% confidence intervals and *p*-values. We evaluated residual spatial autocorrelation after model fitting using (i) the LM residual autocorrelation test for SAR and (ii) Moran’s I for SEM residuals. All analyses were conducted in R (version 4.5.2) using sf, spdep, tmap, and tidyverse.

## Results

Table 1 summarizes descriptive characteristics of the US Deep South counties included in the analysis. Across counties, HIV testing service listing density averaged7.40 sites per 100,000 residents (SD, 8.71), and PGI had a mean of 0.01 (SD, 1.12). PGI was positively correlated with the percentage of non-Hispanic Black residents (*r* = 0.362, *p* < 0.001) and negatively correlated with log household income (*r* = −0.171, *p* < 0.001), social associations (*r* = −0.095, *p* = 0.005), and some college education (*r* = −0.085, *p* = 0.012). Uninsured prevalence (*r* = 0.047, *p* = 0.142) and primary care physicians per 100,000 population (*r* = −0.009, *p* = 0.801) were not significantly correlated with PGI.

**Figure 1:**
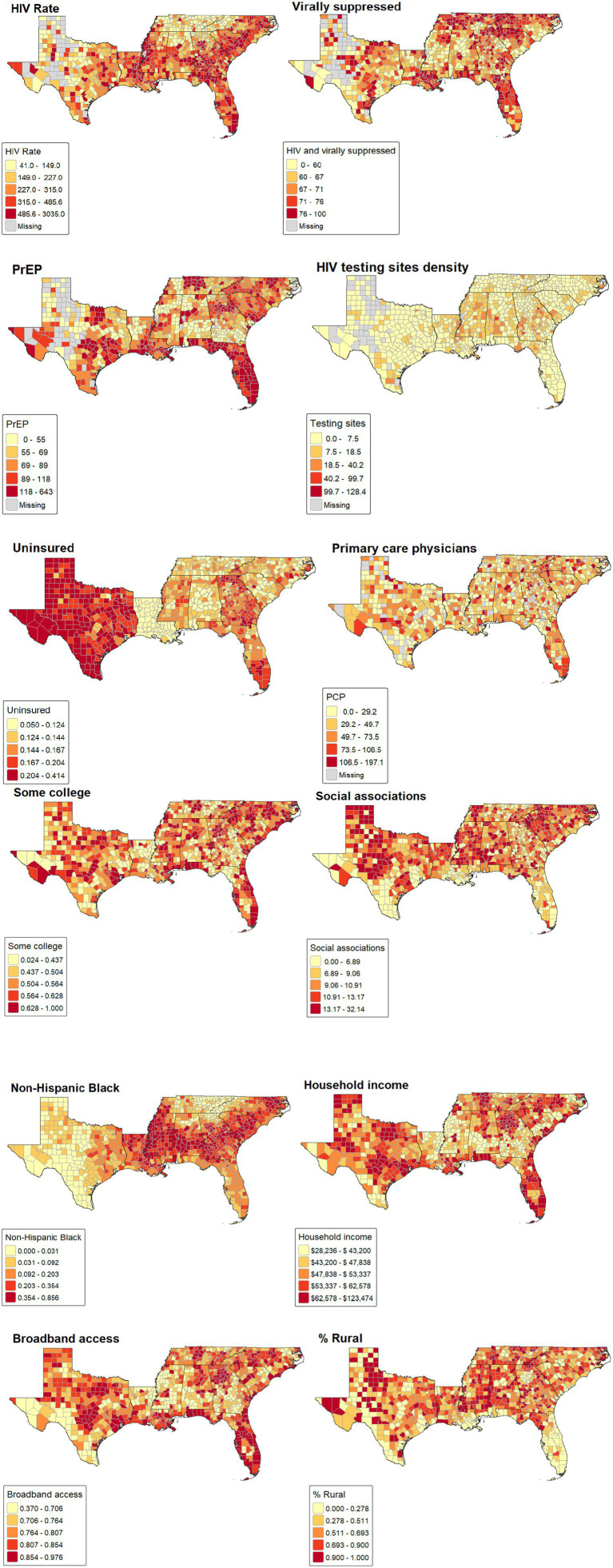
Spatial distribution of the study variables.

Figure 1 highlights the geographical pattern of HIV burden and structural determinants across the US Deep South. Higher HIV prevalence is concentrated in southern Mississippi and the Mississippi Delta, western/central Alabama (including the Black Belt region), and southern Georgia, with additional pockets in eastern Louisiana. Several determinants show similar geographic patterning. These map patterns show that areas of higher HIV burden often overlap with structural disadvantage and limited access-related indicators.

Using the PGI classification, 220 counties were identified as having higher HIV service-planning burden relative to the composite of three measured prevention and care performance proxies (i.e., elevated PGI). We interpret elevated PGI as indicating counties where the geographic concentration of people living with diagnosed HIV is high relative to these available proxy indicators, which may warrant priority programmatic review, rather than as a validated prediction of intervention impact or current transmission intensity.

**Figure 2:**
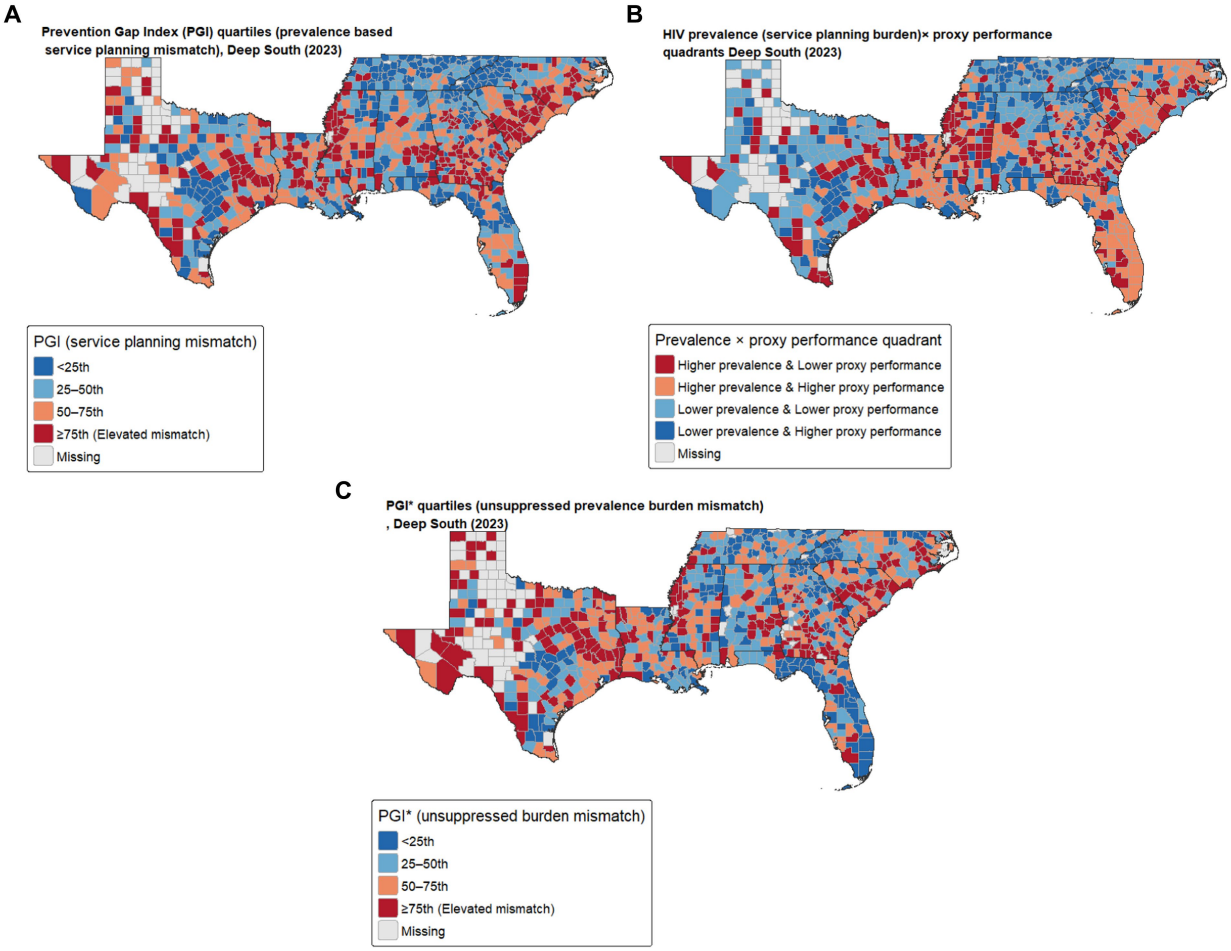
PGI, prevalence-proxy performance quadrants, and PGI* in the US Deep South counties (2023). **(A)** Prevention Gap Index (PGI) quartiles. PGI is defined as standardized HIV prevalence (service-planning burden) minus three standardized prevention and care performance proxies [PrEP utilization, viral suppression, and ln (1 + HIV testing service listing density)]. **(B)** Two-axis classification using median splits of HIV prevalence (service-planning burden) and the proxy performance composite. The “higher prevalence & lower proxy performance” quadrant highlights counties where service-planning burden outpaces measured proxy performance and may warrant priority programmatic review. **(C)** PGI* quartiles using unsuppressed prevalence [prevalence × (1 − viral suppression)] as the burden term and excluding viral suppression from the proxy composite to reduce conceptual overlap between prevalence and suppression.

Because HIV prevalence may reflect survival and historical epidemic dynamics rather than current transmission intensity, we examined PGI* using unsuppressed prevalence as the burden term and excluding viral suppression from the proxy composite. Priority county identification differed meaningfully between PGI and PGI* (top-decile overlap = 65.9%), which indicates that some counties are prioritized primarily under a service-planning burden definition, whereas others are prioritized when burden is defined in a transmission-relevant manner. Across all alternative specifications, top-decile overlap with the baseline PGI ranged from 64.8 to 97.7%, with the largest differences observed under alternative burden (PGI*) and rank-based scoring (Supplementary Table S3). Accordingly, we present PGI (service planning mismatch) and PGI* (unsuppressed burden mismatch) as complementary planning views. These counties were concentrated primarily in Texas, Georgia, Mississippi, Alabama, and South Carolina and were characterized by higher HIV prevalence combined with lower levels of one or more prevention and care performance proxies (PrEP uptake, viral suppression, and/or testing site density). Priority county classification was robust to alternative PGI specifications. To improve interpretability beyond a single standardized difference score, we also classified counties using a two-axis framework that compares HIV prevalence (service-planning burden) and the proxy performance composite (median splits). Counties in the ‘higher prevalence & lower proxy performance’ quadrant represent the most direct mismatch scenario, while the other quadrants distinguish high burden counties with relatively higher proxy performance and lower burden counties with lower proxy performance. Maps of PGI, quadrant classification, and PGI* are shown in Figure 2.

County-level new HIV diagnosis rates (AIDSVu 2023) were available for 564 of 934 US Deep South counties; rates were suppressed/missing for 370 counties, which had a smaller median population than counties with available diagnoses data (20,690 vs. 48,437). In counties with non-missing PGI and diagnosis rates (*n* = 512), PGI was positively associated with new diagnosis rates (Spearman coefficient, *ρ* = 0.512, *p* < 0.001). Median new diagnosis rates increased monotonically across PGI quartiles (Q1 median: 4.5; Q2 11.5; Q3 17.0; Q4 26.0 per 100,000; Kruskal–Wallis *χ*^2^ = 122.1, df = 3, *p* < 0.001). PGI discriminated counties in the top quartile of diagnosis rates (AUC = 0.819), and a 1-SD increase in PGI was associated with higher odds of being a high-diagnosis county (OR, 3.45; 95% CI, 2.53–4.71). Overlap between top-decile PGI counties and top-decile diagnosis counties was 51.9% (Jaccard = 0.35). In the same restricted sample, PGI* was also positively associated with diagnosis rates but weaker (Spearman coefficient, *ρ* = 0.278, *p* < 0.001).

Within the nine-state US Deep South study region, 20 counties were designated as EHE Phase I priority counties and had non-missing PGI values. PGI captured a substantial proportion of these jurisdictions: 65% (13/20) of EHE counties fell within the top quartile of PGI, and 35% (7/20) fell within the top decile. Although EHE counties represented 2.3% of counties with non-missing PGI (20/877), they were overrepresented among high PGI counties (5.9% of the top quartile; 8.0% of the top decile), consistent with convergent validity. PGI values were significantly higher in EHE counties than in non-EHE counties (median PGI 0.77 vs. -0.23, Wilcoxon *p* < 0.001). In logistic regression, each 1-SD increase in PGI was associated with higher odds of EHE designation (OR, 1.41; 95% CI, 1.09–1.80), and PGI demonstrated moderate discrimination for EHE designation (AUC 0.761). Supplementary Table S4 shows each EHE county’s PGI, percentile, and whether it falls in the high PGI top quartile/top decile.

In multivariable ordinary least squares (OLS) regression across 877 US Deep South counties, higher educational attainment (some college education) was associated with lower PGI (*β* = −0.021, *p* < 0.001). In contrast, a higher percentage of non-Hispanic Black residents was associated with higher PGI (*β* = 0.024, *p* < 0.001). Greater rurality was also associated with lower PGI (*β* = −0.011, *p* < 0.001). Broadband access showed a small positive association with PGI (*β* = 0.012, *p* = 0.034). Social associations (*β* = −0.012, *p* = 0.152) and log household income (*β* = −0.265, *p* = 0.225) were not statistically significant in the OLS model. Uninsured prevalence and primary care physician availability were evaluated in preliminary screening analyses but were not retained in the final adjusted models because they were not associated with PGI and did not improve model fit.

Because OLS residuals exhibited spatial dependence, spatial regression models were estimated. Both the spatial lag (SAR) and spatial error (SEM) models improved fit relative to OLS (Akaike Information Criterion [AIC]: OLS, 2043.9; SAR, 2001.1; SEM, 1994.0), with the SEM providing the best overall fit. In the SAR model, the spatial autoregressive parameter was significant (*ρ* = 0.285, *p* < 0.001). Key covariate associations remained directionally consistent: some college education (*β* = −0.020, *p* < 0.001), percentage non-Hispanic Black (*β* = 0.019, *p* < 0.001), broadband access (*β* = 0.013, *p* = 0.016), and rurality (*β* = −0.011, *p* < 0.001); social associations and log income remained non-significant. In the SEM, the spatial error parameter was significant (*λ* = 0.341, *p* < 0.001), indicating residual spatial structure after accounting for observed covariates. The SEM retained strong associations for some college education (*β* = −0.020, *p* < 0.001), percentage of non-Hispanic Black (*β* = 0.024, *p* < 0.001), and rurality (*β* = −0.011, *p* < 0.001), while broadband access was borderline (*β* = 0.011, *p* = 0.059). Residual diagnostics suggested the SEM adequately removed spatial autocorrelation (Moran’s *I* = −0.013, *p* = 0.712) (see Table 2).

**Table 2:** OLS and spatial regression models predicting PGI (US Deep South counties).

Variable	OLS			SAR (lag)			SEM (error)		
	Estimate	95% CI	*p*-value	Estimate	95% CI	*p*-value	Estimate	95% CI	*p*-value
(Intercept)	3.332	−0.885, 7.550	0.121	4.049	−0.006, 8.104	0.050	2.834	−1.633, 7.300	0.214
Some college education	−0.021	−0.028, −0.014	<0.001	−0.020	−0.026, −0.013	<0.001	−0.020	−0.027, −0.013	<0.001
Social association	−0.012	−0.028, 0.004	0.152	−0.008	−0.024, 0.008	0.322	−0.012	−0.029, 0.004	0.151
Non-Hispanic Black	0.024	0.020, 0.027	<0.001	0.019	0.016, 0.023	<0.001	0.024	0.020, 0.028	<0.001
Log household income	−0.265	−0.693, 0.163	0.225	−0.346	−0.758, 0.066	0.100	−0.218	−0.665, 0.229	0.339
Broadband access	0.012	0.001, 0.022	0.034	0.013	0.002, 0.023	0.016	0.011	−0.000, 0.021	0.059
Rural	−0.011	−0.014, −0.009	<0.001	−0.011	−0.013, −0.008	<0.001	−0.011	−0.013, −0.008	<0.001
Spatial parameter				0.285	0.203, 0.367	<0.001	0.341	0.251, 0.432	<0.001
AIC	2043.900			2001.100			1994.000		
BIC	2077.034								
logLik	−1014.950			−991.554			−988.022		
*R*-squared	0.315								
Adjusted *R*-squared	0.310								
*F*-statistic	63.920								
*F p*-value	<0.001								
RMSE	0.809								
Sigma (residual SD)	0.812			0.781			0.775		
LM test *p* (residual autocorrection)				0.130					
Moran’s *I* (residuals)							−0.013		
Moran’s *I p*-value (residuals)							0.712		

OLS p-values are based on *t*-tests; SAR/SEM *p*-values are based on asymptotic *z*-tests. OLS 95% CIs are from lm() confidence intervals; SAR/SEM 95% CIs use normal approximation (estimate ± 1.96*SE). Spatial weights: queen contiguity, row-standardized (style = ‘W’); isolates included with zero.policy = TRUE.

## Discussion

This county-level spatial analysis examined geographic variation in HIV burden and measurable proxies of biomedical prevention and care system performance across the nine US Deep South states. We used the PGI as a descriptive screening summary of the mismatch between HIV prevalence and three planning-relevant proxies aligned with EHE domains: testing service location listing density (Diagnose/access), PrEP utilization (Prevent), and viral suppression (Treat as prevention). As prevalence reflects survival and historical dynamics, PGI should be interpreted as a service planning mismatch measure rather than a predictor of marginal incidence reduction. PGI is a unitless, standardized mismatch measure and should be interpreted as a relative ranking tool rather than an index with intrinsic units.

To address concerns that z-score difference measures can be sensitive to outliers and distributional assumptions, we conducted multiple robustness checks (winsorization and rank-based scoring). We presented a two-axis burden vs. proxy performance classification as an interpretable prioritization rule. Although we used equal weights for transparency and reproducibility, alternative weighting schemes (e.g., expert elicitation or outcome-anchored weights) may be appropriate in specific planning contexts. They should be explored when suitable data are available. Several findings stand out. First, HIV burden remains geographically concentrated, with mapping results showing a pattern of higher HIV prevalence across parts of southern Mississippi and the Mississippi Delta, the Alabama Black Belt/west-central Alabama, southern Georgia, and pockets of eastern Louisiana. Second, in adjusted models, counties with a higher proportion of non-Hispanic Black residents had higher PGI, whereas higher educational attainment (some college education) and greater rurality were associated with lower PGI. Third, spatial regression improved model fitness and reduced residual spatial autocorrelation. This indicates that unmeasured county-level factors are spatially patterned and contribute to prevention gaps (16, 17).

The PGI is intended as a descriptive screening metric to support geographic situational assessment, which identifies counties where measured burden is high relative to available prevention and care proxies. PGI is not designed to estimate causal effects or to quantify the marginal impact of interventions. County-level longitudinal outcomes suitable for predictive validation (e.g., diagnoses/incidence in subsequent years) and independent county-level service coverage measures (e.g., testing volume/positivity, linkage/retention, and PrEP prescriber capacity) were not available consistently across the full nine-state region and timeframe, which limited criterion-based validation in this study. As a partial criterion check, we evaluated PGI against county-level 2023 new diagnosis rates, where non-suppressed, and found that PGI was positively associated with diagnosis rates and discriminated high-diagnosis counties. We therefore emphasize PGI as a tool to prioritize follow-up programmatic assessment using local surveillance and service delivery data not captured here, and we identify external validation using longitudinal outcomes as a key direction for future work.

The PGI framework adds a practical planning layer by identifying counties with high HIV burden relative to prevention and care performance proxies. We identified 220 counties as high need. These counties were concentrated in Georgia (*n* = 55) and Texas (*n* = 52), followed by Mississippi (*n* = 29), Louisiana (*n* = 20), South Carolina (*n* = 20), North Carolina (*n* = 18), Alabama (*n* = 11), Florida (*n* = 11), and Tennessee (*n* = 4). The same counties were mostly identified even when we removed viral suppression or limited viral suppression values to the 5th–95th percentile range before standardization. As viral suppression is linked to prevalence and has both treatment and prevention relevance, we also examined an alternative formulation that incorporates suppression into the burden term as unsuppressed prevalence and removes suppression from the capacity composite. The similarity in priority county identification across PGI and PGI* supports interpretation of the findings as robust to this conceptual specification choice. This robustness matters because it suggests the priority ranking is not driven by any single measure, but by an overall gap across multiple prevention components. The public health value of this approach is consistent with prior spatial epidemiology work arguing that “place-based” HIV planning benefits from summary indicators that align burden with prevention/treatment infrastructure, rather than relying on burden metrics alone (2, 11).

In addition, recent US Deep South relevant spatial work demonstrates how county-to-county heterogeneity can persist even within high burden states, reinforcing the need for geographically precise prioritization (12). These findings suggest that combining HIV burden and prevention resources into one index can help identify counties where prevention investments are most needed to support Ending the HIV Epidemic goals. As an initial external check on validity, PGI showed significant alignment with the federal EHE Phase I priority county list: EHE counties had higher PGI values and were overrepresented among high PGI counties. Because EHE prioritization was based on diagnostic concentration in 2016–2017 and incorporates additional programmatic considerations, incomplete overlap with a 2023 prevalence-based mismatch metric is expected. These findings support PGI as a screening tool to flag counties for programmatic review rather than a definitive targeting rule.

Our findings also reinforce that prevention gaps in the US Deep South are associated with structural conditions. Social associations and educational attainment were protective (lower PGI), aligning with conceptual models in which social connectedness and human capital strengthen navigation of health systems, facilitate diffusion of health information, and support prevention behaviors (18, 19).

The observed association between PGI and the proportion of non-Hispanic Black residents highlights racial variations in HIV burden and prevention access in the US Deep South. National surveillance consistently shows that Black communities bear a disproportionate share of HIV diagnoses, rooted in unequal access to prevention and treatment, stigma, and lower availability of culturally responsive care (3, 8, 20). These results highlight the need for focused prevention strategies that expand PrEP access, strengthen care engagement, and ensure adequate testing infrastructure in counties where structural disadvantage and HIV burden intersect (3, 8, 21, 22).

The association between rurality and lower PGI warrants careful interpretation. A lower PGI in more rural counties does not necessarily imply fewer barriers to HIV prevention. Rather, it may reflect that measured HIV burden is lower relative to prevention and care performance proxies in many rural counties. In contrast, high-mismatch counties remain concentrated in specific rural subregions (e.g., the Delta/Black Belt) where structural disadvantage and HIV burden overlap. This pattern might indicate that rurality is heterogeneous across the US Deep South; prevention gaps may be most pronounced in rural regions rather than along a simple urban–rural gradient. This supports prioritizing rural subregions with concentrated disadvantage (e.g., Delta/Black Belt) rather than applying a uniform rural–urban targeting.

Finally, the spatial model results have direct programmatic meaning. OLS residuals demonstrated spatial autocorrelation, and both spatial lag and spatial error models improved fit, with the spatial error model, performing best and eliminating residual spatial dependence. This suggests that PGI is affected by spatially clustered, unmeasured factors, such as regional service networks, availability of PrEP prescribers, local public health funding, transportation connectivity, stigma climates, and state or local policy, that are not fully captured by measured county covariates. From a public health perspective, this supports using place-based strategies that operate across county boundaries (e.g., regional PrEP access networks, mobile testing, and shared referral systems) rather than relying solely on county-by-county approaches (23–25).

A limitation is that county-level prevention capacity is multidimensional, and our PGI is constrained to indicators consistently available across all counties. The composite does not capture several major, county-relevant prevention levers, including PrEP prescriber supply and clinic capacity, PrEP need/eligibility (e.g., PrEP-to-Need Ratio), testing volume and positivity, linkage to care, retention and ART coverage beyond suppression, STI services, harm reduction (e.g., syringe services), condom distribution, or Ryan White infrastructure. Therefore, PGI should be interpreted as a screening tool that highlights counties where measured burden is high relative to these available proxies and should prompt additional, context-specific programmatic assessment using local surveillance and service delivery data not included here. County new diagnosis rates were suppressed/missing for a substantial share of counties, particularly smaller population counties, which limits the generalizability of restricted-sample validation analyses. In addition, new diagnosis rates are influenced by testing volume, reporting, and diagnosis delays and are not equivalent to modeled incidence.

This study is ecological, so associations at the county level should not be interpreted as individual-level effects or causal relationships. Several indicators rely on surveillance and administrative data and may be affected by reporting completeness and measurement error. In particular, the HIV.gov locator-derived testing measure reflects the density of listed testing service locations and does not capture hours of operation, staffing, availability of rapid testing, whether sites are accepting clients, outreach/mobile testing, or testing throughput/volume. It also does not account for cross-county travel patterns, which may be especially important in rural areas where residents may access services outside their county of residence. Some counties, especially in Texas, were excluded due to missing HIV burden data, which may limit generalizability within the region. Finally, PGI weighs each prevention component equally; alternative weighting schemes may be appropriate in specific planning contexts and should be explored in future work. Despite these limitations, PGI highlights priority counties where the burden outpaces prevention resources.

### Public health implications

The results support prioritizing programmatic review and planning in counties identified as high need by PGI (high burden relative to measured prevention and care proxies), with emphasis on (1) scaling PrEP access and prescribing capacity, (2) strengthening retention in care and viral suppression through linkage and re-engagement strategies, and (3) improving testing availability in underserved areas. Integrated service models that combine HIV testing, STI screening/treatment, and rapid linkage-to-care may outperform fragmented programs by reducing missed opportunities across the prevention and care continuum. Geographic-access research has shown that many areas, especially in Southern contexts, face limited access to PrEP providers and longer travel times to prevention services (13), which reinforces the need for mobile, decentralized, and community-based delivery models. Using PGI as a monitoring tool can also help track whether prevention and care performance proxies are improving relative to burden over time and can support transparent, data-driven resource allocation aligned with Ending the HIV Epidemic goals.

## Data Availability

The data used in this study were obtained from a combination of publicly available and third-party sources. Publicly available data can be accessed from the original sources cited in the article. HIV testing center location data are available from HIV.gov upon request. Additional data accessed through PolicyMap are subject to subscription and licensing restrictions. Therefore, the compiled dataset used for this study is not publicly available from the authors; requests for restricted source data should be directed to the original data providers.
