# Supplementary material for: A county-level HIV prevention gap index in the US Deep South using publicly available proxy indicators
Source: Front Public Health. 2026 Apr 13;14:1793411. doi: 10.3389/fpubh.2026.1793411 (PMC13111552; doi:10.3389/fpubh.2026.1793411)
Supplement: Supplementary file 1 [file Table_1.docx]

**Supplementary Table S1**

| **Variables** | **Description** | **Source** |
| --- | --- | --- |
| ***HIV and Prevention*** |  |  |
| HIV 2023 | Rate of people living with an HIV diagnosis per 100,000 people in 2023. | [7, 14] |
| PrEP 2023 | PrEP use rate (per 100,000 adults aged ≥16 years), 2023 | [7] |
| Viral Suppression | Percent of persons aged ≥13 years living with diagnosed HIV who were virally suppressed (≤200 copies/mL) at most recent viral load test, 2023 | [7] |
| HIV testing service listing density (locator-based) | Listed HIV testing service locations per 100,000 residents (derived from HIV.gov locator; county aggregation); proxy for potential geographic availability. | [16] |
| ***Health Access*** |  |  |
| Uninsured | Percentage of adults under age 65 without health insurance. The 2025 Annual Data Release used data from 2022 for this measure. | [15] |
| Primary Care Physicians | Primary care physicians per 100,000 population (2021). | [15] |
| ***Socioeconomic Factors*** |  | [15] |
| Some College | Percentage of adults ages 25-44 with some post-secondary education. The 2025 Annual Data Release used data from 2019-2023 for this measure. | [15] |
| Social Associations | Number of membership associations per 10,000 population. The 2025 Annual Data Release used data from 2022 for this measure. | [15] |
| Non-Hispanic Black | Percentage of population identifying as non-Hispanic Black or African American. The 2025 Annual Data Release used data from 2023 for this measure. | [15] |
| Household Income | The income where half of households in a county earn more and half of households earn less. The 2025 Annual Data Release used data from 2023 & 2019-2023 for this measure. | [15] |
| Rural | Percentage of population living in a census-defined rural area, 2023 | [15] |
