# Supplementary material for: A county-level HIV prevention gap index in the US Deep South using publicly available proxy indicators
Source: Front Public Health. 2026 Apr 13;14:1793411. doi: 10.3389/fpubh.2026.1793411 (PMC13111552; doi:10.3389/fpubh.2026.1793411)
Supplement: Supplementary file 2 [file Table_2.docx]

**Supplementary Table S2.** Conceptual mapping of PGI components to HIV planning domains

| **Planning domain (EHE/continuum)** | **Indicator used in PGI** | **What it captures (why relevant)** | **What it does not capture (what is missing)** |
| --- | --- | --- | --- |
| Diagnose / service access | Testing service listing density (locator-based): ln (1 + sites per 100k) | Geographic availability of listed testing locations; proxy for potential access | Testing volume/throughput, hours, staffing, mobile/outreach testing, positivity, acceptability, cross county travel |
| Prevent (biomedical prevention delivery) | PrEP use rate | Uptake of PrEP at county level; proxy for penetration of PrEP delivery | PrEP eligibility/need (e.g., PrEP-to-Need Ratio), prescriber capacity, affordability, adherence/persistence, stigma |
| Treat (treatment as prevention) | Viral suppression (%) among PLWH with diagnosed HIV | Care performance relevant to transmission risk; core EHE Treat metric | Linkage/retention details, ART coverage, care quality, time-to-suppression; surveillance completeness |
