# Supplementary material for: A county-level HIV prevention gap index in the US Deep South using publicly available proxy indicators
Source: Front Public Health. 2026 Apr 13;14:1793411. doi: 10.3389/fpubh.2026.1793411 (PMC13111552; doi:10.3389/fpubh.2026.1793411)
Supplement: Supplementary file 3 [file Table_3.docx]

**Supplementary Table S3.** Overlap of high need county identification across PGI specifications (baseline vs alternatives)

| **Specification (alternative PGI construction)** | **Rationale** | **Top decile overlap with baseline (%)** |
| --- | --- | --- |
| Exclude viral suppression from capacity | Assesses sensitivity to including viral suppression in the proxy performance composite | 84.1 |
| Winsorize viral suppression only (5th-95th) prior to z-scoring | Reduces influence of extreme viral suppression values on z-scores/outlier sensitivity | 97.7 |
| PGI* using unsuppressed prevalence as burden; capacity excludes viral suppression | Addresses potential conceptual overlap between prevalence burden and suppression; aligns burden with treatment as prevention logic | 65.9 |
| Winsorize all components (5th-95th) prior to z-scoring | Tests overall outlier sensitivity of z-score difference index across all components | 90.9 |
| Rank based PGI (percentile ranks for all components) | Tests robustness to distributional assumptions of z-scores; reduces influence of scale/outliers | 64.8 |
| PrEP heavy weights (0.50/0.25/0.25) | Assesses sensitivity to alternative weighting emphasizing PrEP utilization in the proxy performance composite | 91.9 |
| Viral suppression heavy weights (0.25/0.50/0.25) | Assesses sensitivity to alternative weighting emphasizing viral suppression in the proxy performance composite | 90.7 |
| Testing listing heavy weights (0.25/0.25/0.50) | Assesses sensitivity to alternative weighting emphasizing testing service listing density in the proxy performance composite | 89.5 |
