# Supplementary material for: A county-level HIV prevention gap index in the US Deep South using publicly available proxy indicators
Source: Front Public Health. 2026 Apr 13;14:1793411. doi: 10.3389/fpubh.2026.1793411 (PMC13111552; doi:10.3389/fpubh.2026.1793411)
Supplement: Supplementary file 4 [file Table_4.docx]

**Supplementary Table S4.** EHE Phase I priority counties in the study region and PGI values (2023).

| **State** | **County** | **County FIPS (GEOID)** | **PGI** | **PGI percentile** | **High PGI (top quartile)** | **High PGI (top decile)** |
| --- | --- | --- | --- | --- | --- | --- |
| GA | Fulton County | 13121 | 3.08 | 98.3 | Yes | Yes |
| GA | DeKalb County | 13089 | 2.93 | 98.2 | Yes | Yes |
| LA | East Baton Rouge Parish | 22033 | 2.02 | 96.5 | Yes | Yes |
| LA | Orleans Parish | 22071 | 1.92 | 96 | Yes | Yes |
| TN | Shelby County | 47157 | 1.83 | 95.3 | Yes | Yes |
| TX | Dallas County | 48113 | 1.17 | 91 | Yes | Yes |
| TX | Harris County | 48201 | 1.14 | 90.6 | Yes | Yes |
| FL | Miami-Dade County | 12086 | 1.03 | 88.6 | Yes | No |
| GA | Cobb County | 13067 | 0.868 | 86.4 | Yes | No |
| FL | Broward County | 12011 | 0.811 | 85.7 | Yes | No |
| FL | Duval County | 12031 | 0.729 | 84.1 | Yes | No |
| NC | Mecklenburg County | 37119 | 0.44 | 77.6 | Yes | No |
| FL | Palm Beach County | 12099 | 0.378 | 75.7 | Yes | No |
| FL | Hillsborough County | 12057 | 0.25 | 71.1 | No | No |
| GA | Gwinnett County | 13135 | 0.241 | 70.7 | No | No |
| TX | Bexar County | 48029 | 0.081 | 64.5 | No | No |
| TX | Tarrant County | 48439 | -0.032 | 59 | No | No |
| FL | Pinellas County | 12103 | -0.109 | 55.5 | No | No |
| FL | Orange County | 12095 | -0.656 | 25.7 | No | No |
| TX | Travis County | 48453 | -1.95 | 0.342 | No | No |
